# Effects of systemic oxytocin and beta-3 receptor agonist (CL 316243) treatment on body weight and adiposity in male diet-induced obese rats

**DOI:** 10.1101/2024.09.27.615550

**Published:** 2024-09-30

**Authors:** Jared D. Slattery, June R. Rambousek, Edison Tsui, Mackenzie K. Honeycutt, Matvey Goldberg, James L. Graham, Tomasz A. Wietecha, Tami Wolden-Hanson, Kevin D. O’Brien, Peter J. Havel, James E. Blevins

**Affiliations:** 1 VA Puget Sound Health Care System, Office of Research and Development Medical Research Service, Department of Veterans Affairs Medical Center, Seattle, WA 98108, USA; 2 Division of Metabolism, Endocrinology and Nutrition, Department of Medicine, University of Washington School of Medicine, Seattle, WA 98195, USA; 3 Division of Cardiology, Department of Medicine, University of Washington School of Medicine, Seattle, WA 98195, USA; 4 UW Medicine Diabetes Institute, University of Washington School of Medicine, Seattle, WA 98109; 5 Department of Nutrition, University of California, Davis, CA 95616, USA; 6 Department of Molecular Biosciences, School of Veterinary Medicine, University of California, Davis, CA 95616, USA

**Keywords:** Obesity, brown adipose tissue, white adipose tissue, food intake, oxytocin

## Abstract

Previous studies have implicated hindbrain oxytocin (OT) receptors in the control of food intake and brown adipose tissue (BAT) thermogenesis. We recently demonstrated that hindbrain [fourth ventricle (4V)] administration of oxytocin (OT) could be used as an adjunct to drugs that directly target beta-3 adrenergic receptors (β3-AR) to elicit weight loss in diet-induced obese (DIO) rodents. What remains unclear is whether systemic OT can be used as an adjunct with the β3-AR agonist, CL 316243, to increase BAT thermogenesis and elicit weight loss in DIO rats. We hypothesized that systemic OT and β3-AR agonist (CL 316243) treatment would produce an additive effect to reduce body weight and adiposity in DIO rats by decreasing food intake and stimulating BAT thermogenesis. To test this hypothesis, we determined the effects of systemic (subcutaneous) infusions of OT (50 nmol/day) or vehicle (VEH) when combined with daily systemic (intraperitoneal) injections of CL 316243 (0.5 mg/kg) or VEH on body weight, adiposity, food intake and brown adipose tissue temperature (T_IBAT_). OT and CL 316243 monotherapy decreased body weight by 8.0±0.9% (*P*<0.05) and 8.6±0.6% (*P*<0.05), respectively, but OT in combination with CL 316243 produced more substantial weight loss (14.9±1.0%; *P*<0.05) compared to either treatment alone. These effects were associated with decreased adiposity, energy intake and elevated T_IBAT_ during the treatment period. The findings from the current study suggest that the effects of systemic OT and CL 316243 to elicit weight loss are additive and appear to be driven primarily by OT-elicited changes in food intake and CL 316243-elicited increases in BAT thermogenesis.

## Introduction

The obesity epidemic and its associated complications, increase the risk for cardiovascular disease (CVD), hypertension, cancer, type 2 diabetes, and COVID-19 [1; 2]. Many of the monotherapies to treat obesity are of limited effectiveness, associated with adverse and/or unwanted side effects (i.e. diarrhea, nausea, vomiting, sleep disturbance and depression) and/or are poorly tolerated. Improvements have been made in monotherapies to treat obesity, particularly within the family of drugs that target the glucagon-like peptide-1 receptor (GLP-1R). While the FDA recently approved the use of the long-acting and highly effective GLP-1R agonist, semaglutide [3], it can also be associated with mild to moderate gastrointestinal (GI) side effects [3; 4], thus highlighting the need for continued optimization of existing treatments.

Recent studies suggest that combination therapy (co-administration of different compounds) and monomeric therapy (dual or triple agonists in single molecule) are more effective than monotherapy for prolonged weight loss [5; 6]. Marked weight loss has been reported in long-term (20 weeks to ≥ 1 year) clinical studies in humans treated with the amylin analogue, cagrilintide, and semaglutide (≈ 15.6 to 17.1% of initial body weight [7; 8]) and the FDA-approved drug, Qsymia (topiramate + phentermine) (≈ 10.9% of initial body weight; [9]). Alternatively, the monomeric compound, tirzepatide (Zepbound^™^), targets both GLP-1R and glucose-dependent insulinotropic polypeptide (GIP), and was recently reported to elicit 20.9% and 25.3% weight loss in humans with obesity over 72- [10] and 88-week trials [11]. In addition, retatrutide, a triple-agonist that targets GIP, glucagon receptors (GCGR) and GLP-1R was reported to reduce body weight by 24.2% over a 48-week trial [12]. Despite the considerable improvements that have been made with respect to weight loss, these treatments are still associated with adverse GI side effects [10], leading, in some cases, to the discontinuation of the drug in up to 7.1% of participants [10].

While the hypothalamic neuropeptide, oxytocin (OT) is largely associated with reproductive behavior [13], recent studies implicate an important role for OT in the regulation of body weight [14; 15; 16; 17]. Studies to date indicate that OT elicits weight loss, in part, by reducing food intake and increasing lipolysis [18; 19; 20] and energy expenditure [18; 21; 22; 23]. While OT is effective at evoking prolonged weight loss in DIO rodents [19; 22; 23; 24; 25; 26; 27; 28] and nonhuman primates [18], its overall effectiveness as a monotherapy to treat obesity is relatively modest following 4–8 week treatments in DIO mice (≈4.9%) [28], rats (≈8.7%) [28] and rhesus monkeys (≈3.3%) [18] thus making it more suited as a combination therapy with other drugs that work through other mechanisms. Head and colleagues recently reported that systemic OT and the opioid antagonist, naltrexone, resulted in an enhanced reduction of high-fat, high-sugar meal in rats [29]. Recently, we found that hindbrain (fourth ventricle; 4V) OT treatment in combination with systemic treatment with CL-316243, a drug that directly targets beta-3 adrenergic receptors (β3-AR) to increase BAT thermogenesis [28; 30; 31; 32; 33], resulted in greater weight loss (15.5 ± 1.2% weight loss) than either OT (7.8 ± 1.3% weight loss) or CL 316243 (9.1 ± 2.1% weight loss) alone [34].

The goal of the current study was to test if systemic OT treatment could be used as an adjunct with the β3-AR agonist, CL 316243, to increase BAT thermogenesis and elicit weight loss in DIO rats when using a more translational route of administration for OT delivery. We hypothesized that systemic OT and β3-AR agonist (CL 316243) treatment would produce an additive effect to reduce body weight and adiposity in DIO rats by decreasing food intake and stimulating BAT thermogenesis. To test this, we determined the effects of systemic (subcutaneous) infusions of OT (50 nmol/day) or vehicle (VEH) when combined with daily systemic (intraperitoneal) injections of CL 316243 (0.5 mg/kg) or VEH on body weight, adiposity, food intake, brown adipose tissue temperature (T_IBAT_) and thermogenic gene expression.

## Methods

### Animals

Adult male Long-Evans rats [~ 8–9 weeks old, 292–349 grams at start of high fat dietary (HFD) intervention/~ 8–10 months old, 526–929 g body weight at study onset] were initially obtained from Envigo (Indianapolis, IN) and maintained for at least 4 months on a high fat diet (HFD) prior to study onset. All animals were housed individually in Plexiglas cages in a temperature-controlled room (22±2°C) under a 12:12-h light-dark cycle. All rats were maintained on a 1 a.m./1 p.m. light cycle. Rats had *ad libitum* access to water and a HFD providing 60% kcal from fat (approximately 6.8% kcal from sucrose and 8.9% of the diet from sucrose) (Research Diets, D12492, New Brunswick, NJ). The research protocols were approved both by the Institutional Animal Care and Use Committee of the Veterans Affairs Puget Sound Health Care System (VAPSHCS) and the University of Washington in accordance with NIH’s *Guide for the Care and Use of Laboratory Animals* (NAS, 2011) [35].

### Drug Preparation

Fresh solutions of OT acetate salt (Bachem Americas, Inc., Torrance, CA) were solubilized in sterile water and subsequently primed in sterile 0.9% saline at 37° C for approximately 40 hours prior to minipump implantation based on manufacturer’s recommended priming instructions for ALZET^®^ model 2004 minipumps. CL 316243 (Tocris/Bio-Techne Corporation, Minneapolis, MN) was solubilized in sterile water each day of each experiment.

### Subcutaneous implantations of osmotic minipumps

Animals were implanted with an osmotic minipump (model 2004, DURECT Corporation Cupertino, CA) one week prior to CL 316343 treatment as previously described [34].

### Implantation of temperature transponders underneath IBAT

Rats were anesthetized with isoflurane prior to having the dorsal surface along the upper midline of the back shaved and scrubbed with 70% ethanol followed by betadine swabs as previously described [28]. Following an incision (1 “) along the midline of the interscapular area a temperature transponder (14 mm long/2 mm wide) (HTEC IPTT-300; Bio Medic Data Systems, Inc., Seaford, DE) was implanted underneath the left IBAT pad as previously described [28; 36; 37]. The transponder was subsequently secured in place by suturing it to the brown fat pad with sterile silk suture. The interscapular incision was closed with Nylon sutures (5–0), which were removed in awake animals approximately 10–14 days post-surgery. HTEC IPTT-300 transponders were used in place of IPTT-300 transponders to enhance accuracy in our measurements as previously described [28].

### Acute IP injections and measurements of T_IBAT_

CL 316243 (or saline vehicle; 0.1 ml/kg injection volume) was administered immediately prior to the start of the dark cycle following 4 hours of food deprivation. Animals remained without access to food for an additional 1 (**Study 2–3**) or 4 h (**Study 1**) during the course of the T_IBAT_ measurements. A handheld reader (DAS-8007-IUS Reader System; Bio Medic Data Systems, Inc.) was used to collect measurements of T_IBAT_.

### Body Composition

Determinations of lean body mass and fat mass were made on un-anesthetized rats by quantitative magnetic resonance using an EchoMRI 4-in-1–700^™^ instrument (Echo Medical Systems, Houston, TX) at the VAPSHCS Rodent Metabolic Phenotyping Core. Measurements were taken prior to 4V cannulations and minipump implantations as well as at the end of the infusion period.

## Study Protocols

### Study 1: Determine the dose-response effects of systemic (SC) infusion of OT (16 and 50 nmol/day) on body weight, adiposity and energy intake in DIO rats.

Rats were fed *ad libitum* and maintained on HFD for approximately 5.5 months prior to prior to being implanted with a temperature transponder underneath the left IBAT depot. Rats were subsequently maintained on a daily 4-h fast and received minipumps to infuse vehicle or OT (16 or 50 nmol/day) over 29 days. These doses was selected based on a dose of OT found to be effective at reducing body weight when administered subcutaneously [19] or into the 4V [38] of DIO rats. Daily food intake and body weight were also tracked for 29 days.

### Study 2: Effect of chronic 4V OT infusions (16 nmol/day) and systemic beta-3 receptor agonist (CL 31643) administration (0.5 mg/kg) on body weight, body adiposity, energy intake and T_IBAT_ in male DIO rats.

Rats (~ 10 mo old; 526–929 g at start of study) were fed *ad libitum* and maintained on HFD for approximately 7.5 months prior to receiving implantations of temperature transponders underneath the left IBAT pad in addition to 4V cannulas and 28-day minipumps to infuse vehicle or OT (16 nmol/day) over 28 days, respectively. After having matched animals for OT-elicited reductions in body weight (infusion day 7), DIO rats subsequently received 1x daily IP injections of VEH or CL 316243 (0.5 mg/kg). We selected this dose because it elevated T_IBAT_ at doses that failed to produce elevations in heart rate in lean rats [39]. In addition, this dose produced comparable weight loss to that of OT alone in **Study 2**. T_IBAT_ was measured daily at baseline (−4 h; 9:00 a.m.), immediately prior to IP injections (0 h; 12:45–1:00 p.m.), and at 0.25, 0.5, 0.75, 1, 20 and 24-h post-injection. In addition, daily food intake and body weight were also tracked for 28 days. Data from animals that received the single dose of CL-31643 were analyzed over the 28-day infusion period.

### Adipose tissue processing for adipocyte size and UCP-1 analysis

IWAT and EWAT depots were collected at the end of the infusion period in rats from **Study 2**. Rats from each group were euthanized following a 3-h fast. Rats were euthanized with intraperitoneal injections of ketamine cocktail [ketamine hydrochloride (214.3 mg/kg), xylazine (10.71 mg/kg) and acepromazine (3.3 mg/kg) in an injection volume up to 2 mL/rat] and transcardially exsanguinated with PBS followed by perfusion with 4% paraformaldehyde in 0.1 M PBS. Adipose tissue (IBAT, IWAT, and EWAT) was dissected and placed in 4% paraformaldehyde-PBS for 24 h and then placed in 70% ethanol (EtOH) prior to paraffin embedding. Sections (5 μm) sampled were obtained using a rotary microtome, slide-mounted using a floatation water bath (37°C), and baked for 30 min at 60°C to give approximately 15–16 slides/fat depot with two sections/slide.

### Adipocyte size analysis and UCP-1 staining

Adipocyte size analysis was performed on deparaffinized and digitized IWAT and EWAT sections. The average cell area from two randomized photomicrographs was determined using the built-in particle counting method of ImageJ software (National Institutes of Health, Bethesda, MD). Fixed (4% PFA), paraffin-embedded adipose tissue was sectioned and stained with a primary rabbit anti-UCP-1 antibody (1:100; Abcam, Cambridge, MA (#ab10983/RRID: AB_2241462)] as has been previously described in lean C57BL/6J mice [40] and both lean and DIO C57BL/6 mice after having been screened in both IBAT and IWAT of Ucp1^+/−^ and Ucp1^−/−^ mice [41]. Immunostaining specificity controls included omission of the primary antibody and replacement of the primary antibody with normal rabbit serum at the same dilution as the respective primary antibody. Area quantification for UCP1 staining was performed on digital images of immunostained tissue sections using image analysis software (Image Pro Plus software, Media Cybernetics, Rockville, MD, USA). Slides were visualized using bright field on an Olympus BX51 microscope (Olympus Corporation of the Americas; Center Valley, PA) and photographed using a Canon EOS 5D SR DSLR (Canon U.S.A., Inc., Melville, NY) camera at 100X magnification. Values for each tissue within a treatment were averaged to obtain the mean of the treatment group.

### Blood collection

Blood was collected from 4-h (**Study 1**) or 6-h fasted rats (**Study 2**) within a 2-h window towards the end of the light cycle (10:00 a.m.−12:00 p.m.) as previously described in DIO CD^®^ IGS rats and mice [24; 28]. Animals from **Study 2** were euthanized at 2-h post-CL 316243 or VEH treatment. Treatment groups were counterbalanced at time of euthanasia to avoid time of day bias. Blood samples [up to 3 mL] were collected immediately prior to transcardial perfusion by cardiac puncture in chilled K2 EDTA Microtainer Tubes (Becton-Dickinson, Franklin Lakes, NJ). Whole blood was centrifuged at 6,000 rpm for 1.5-min at 4°C; plasma was removed, aliquoted and stored at −80°C for subsequent analysis.

### Plasma hormone measurements

Plasma leptin and insulin were measured using electrochemiluminescence detection [Meso Scale Discovery (MSD^®^), Rockville, MD] using established procedures [28; 42]. Intra-assay coefficient of variation (CV) for leptin was 2.7% and 3.2% for insulin. The range of detectability for the leptin assay is 0.137–100 ng/mL and 0.069–50 ng/mL for insulin. Plasma fibroblast growth factor-21 (FGF-21) (R&D Systems, Minneapolis, MN) and irisin (AdipoGen, San Diego, CA) levels were determined by ELISA. The intra-assay CV for FGF-21 and irisin were 4.5% and 8.4%, respectively; the ranges of detectability were 31.3–2000 pg/mL (FGF-21) and 0.078–5 μg/mL (irisin). Plasma adiponectin was also measured using electrochemiluminescence detection Meso Scale Discovery (MSD^®^), Rockville, MD] using established procedures [28; 42]. Intra-assay CV for adiponectin was 1.1%. The range of detectability for the adiponectin assay is 2.8–178 ng/mL. The data were normalized to historical values using a pooled plasma quality control sample that was assayed in each plate.

### Blood glucose and lipid measurements

Blood was collected for glucose measurements by tail vein nick following a 4 (**Study 1**) or 6-h fast (**Study 2**) and measured with a glucometer using the AlphaTRAK 2 blood glucose monitoring system (Abbott Laboratories, Abbott Park, IL) [43]. Tail vein glucose was measured at 2-h post-CL 316243 or VEH treatment (**Study 2**). Total cholesterol (TC) [Fisher Diagnostics (Middletown, VA)] and free fatty acids (FFAs) [Wako Chemicals USA, Inc., Richmond, VA)] were measured using an enzymatic-based kits. Intra-assay CVs for TC and FFAs were 1.4 and 2.3%, respectively. These assay procedures have been validated for rodents [44].

### Tissue collection for quantitative real-time PCR (qPCR)

IBAT and IWAT tissue was collected from 4 (**Study 1**) or 6-h fasted rats (**Study 2**). In addition, animals from **Study 2** were euthanized at 2-h post-CL 316243 (0.5 mg/kg) or VEH administration. IBAT and IWAT were collected within a 2-h window towards the end of the light cycle (10:00 a.m.−12:00 p.m.) as previously described in DIO CD^®^ IGS/Long-Evans rats and C57BL/6J mice [24; 28; 38]. Tissue was rapidly removed, wrapped in foil and frozen in liquid N2. Samples were stored frozen at −80°C until analysis.

### qPCR

RNA extracted from samples of IBAT and IWAT (Studies 1–2) were analyzed using the RNeasy Lipid Mini Kit (Qiagen Sciences Inc, Germantown, MD) followed by reverse transcription into cDNA using a high-capacity cDNA archive kit (Applied Biosystems, Foster City, CA). Quantitative analysis for relative levels of mRNA in the RNA extracts was measured in duplicate by qPCR on an Applied Biosystems 7500 Real-Time PCR system (Thermo Fisher Scientific, Waltham, MA) and normalized to the cycle threshold value of Nono mRNA in each sample. The TaqMan^®^ probes used in the study were Thermo Fisher Scientific Gene Expression Assay probes. The probe for rat *Nono* (Rn01418995_g1), UCP-1 (*Ucp1*; catalog no. Rn00562126_m1), beta 1 adrenergic receptor (β1-AR) (*Adrb1*; catalog no. Rn00824536_s1), β3-AR (*Adrb3*; catalog no. Rn01478698_g1), type 2 deiodinase (D2) (*Dio2*; catalog no. Rn00581867_m1), G-protein coupled receptor 120 (*Gpr120*; catalog no. Rn01759772_m1), cell death-inducing DNA fragmentation factor α-like effector A (*Cidea*; catalog no. Rn04181355_m1), peroxisome proliferator-activated receptor gamma coactivator 1α (*Ppargc1a*; catalog no. Rn00580241_m1) and PR domain containing 16 (*Prdm16*; catalog no. Rn01516224_m1) were acquired from Thermo Fisher Scientific. Relative amounts of target mRNA were determined using the Comparative C_T_ or 2-^ΔΔC^_T_ method [45] following adjustment for the housekeeping gene. Specific mRNA levels of all genes of interest were normalized to the cycle threshold value of Nono mRNA in each sample and expressed as changes normalized to controls (vehicle/vehicle treatment).

### Transponder placement

All temperature transponders were confirmed to have remained underneath the IBAT depot at the conclusion of the study.

### Statistical Analyses

All results are expressed as means ± SE. Comparisons between multiple groups involving between subjects designs were made using one- or two-way ANOVA as appropriate, followed by a post-hoc Fisher’s least significant difference test. Comparisons involving within-subjects designs were made using a one-way repeated-measures ANOVA followed by a post-hoc Bonferroni Test. Analyses were performed using the statistical program SYSTAT (Systat Software, Point Richmond, CA). Differences were considered significant at *P*<0.05, 2-tailed.

## Results

### Study 1: Determine the dose-response effects of systemic (SC) infusion of OT (16 and 50 nmol/day) on body weight, adiposity and energy intake in DIO rats

**Figure 1A-D:**
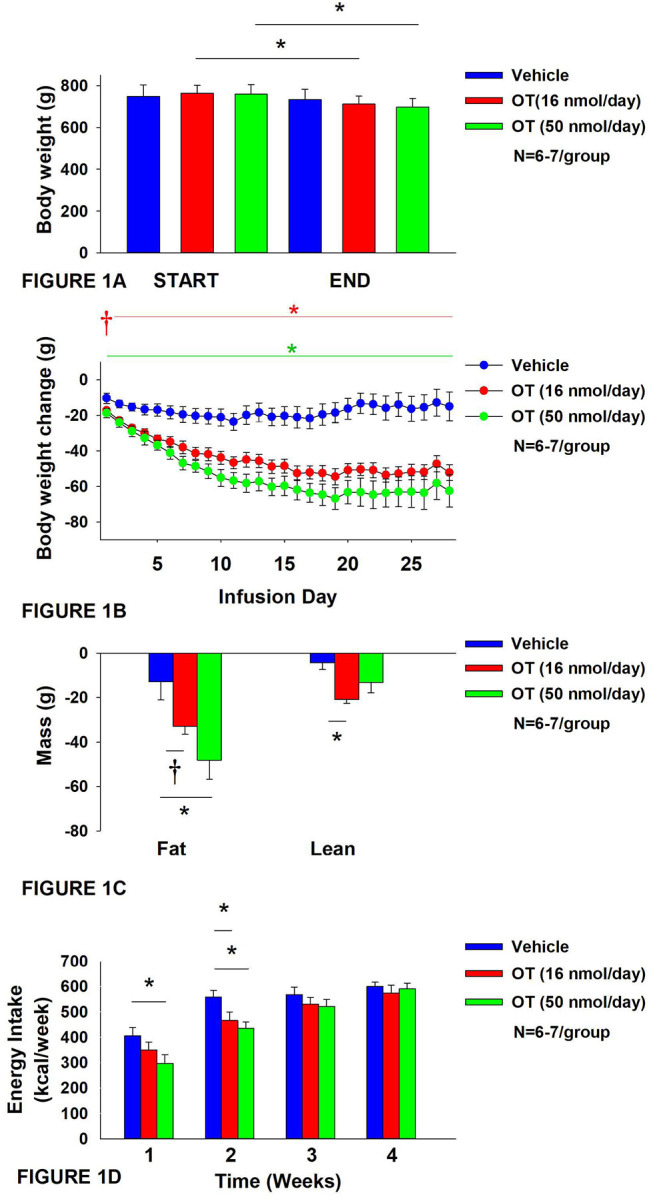
Determine the dose-response effects of systemic (SC) infusion of OT (16 and 50 nmol/day) on body weight, adiposity and energy intake in DIO rats. *A*, Rats were maintained on HFD (60% kcal from fat; N=6–7/group) for approximately 5.5 months prior to being implanted with temperature transponders and allowed to recover for 1–2 weeks prior to being implanted with subcutaneous minipumps. *A*, Effect of chronic subcutaneous OT or vehicle on body weight in DIO rats; *B*, Effect of chronic subcutaneous OT or vehicle on body weight change in DIO rats; *C*, Effect of chronic subcutaneous OT or vehicle on adiposity in DIO rats; *D*, Effect of chronic subcutaneous OT or vehicle on adiposity in DIO rats. Data are expressed as mean ± SEM. *P<0.05, †0.05<*P*<0.1 OT vs. vehicle.

Prior to treatment, groups were matched for body weight and adiposity (vehicle: 749±50 grams; OT (16 nmol/day): 763.6±37.6 grams/42±2.6% fat; OT (50 nmol/day): 759±45.4 grams/39.2±1.6%. There was no difference in body weight [(F(2,17) = 0.027, *P*=NS)] or percent adiposity [(F(2,17) = 0.003, *P*=NS)] between groups prior to treatment onset. As expected, body weight of DIO rats remained stable over the month of vehicle treatment relative to pre-treatment [(F(1,5) = 2.865, *P*=0.151)] (Figure 1A). In contrast to vehicle treatment, systemic OT (16 nmol/day) resulted in a significant reduction of body weight relative to OT pre-treatment [(F(1,6) = 140.799, *P*<0.01)] (Figure 1A; *P*<0.05). Furthermore, SC OT, at a 3-fold higher dose (50 nmol/day), also resulted in a significant elevation of body weight relative to pre-treatment [(F(1,6) = 47.271, *P*<0.01)].

In addition, SC OT (16 and 50 nmol/day) was able to reduce weight gain (Figure 1B) relative to vehicle treatment throughout the 28-day infusion period. SC OT (50 nmol/day), at a dose that was at least 3-fold higher than the centrally effective dose (16 nmol/day), reduced weight gain throughout the entire 28-day infusion period. SC OT (16 nmol/day) treated rats had reduced weight gain between days 2–29 (*P*<0.05) while SC OT (50 nmol/day) reduced weight gain between days 1–29 (*P*<0.05). There was an overall effect of OT to reduce relative fat mass (pre- vs post-intervention) [(F(2,17) = 6.052, *P*=0.010)]. SC OT (50 nmol/day) reduced fat mass (*P*<0.05) and there was also a tendency for the lower dose (16 nmol/day) to reduce relative fat mass (*P*=0.066) (Figure 1C; *P*<0.05).

There was also an overall effect of OT to reduce relative lean mass (pre- vs post-intervention) [(F(2,17) = 5.572, *P*=0.014)]. Specifically, SC OT (16 nmol/day) reduced relative lean mass at the lower dose (16 nmol/day; *P*<0.01) while the higher dose (50 nmol/day) tended to reduce relative lean mass (*P*=0.090). Note that there was no significant reduction in total fat mass or lean mass (*P*=NS).

The changes in body weight and relative fat mass were not associated with any changes in plasma leptin, insulin, glucose or total cholesterol (Table 1). These effects that were mediated, at least in part, by a modest reduction of energy intake that was apparent during weeks 1 (50 nmol/day) and 2 (16 and 50 nmol/day) of OT treatment (Figure 1D; *P*<0.05).

### Study 2: Effect of chronic 4V OT infusions (16 nmol/day) and systemic β3-AR agonist (CL 31643) administration (0.5 mg/kg) on body weight, body adiposity and energy intake in male DIO rats.

The goal of this study was to determine the effects of chronic OT treatment in combination with a single dose (identified in **Study 1**) of the β3-AR agonist, CL 316243, on body weight and adiposity in DIO rats. By design, DIO rats were obese as determined by both body weight (804±14 g) and adiposity (310±11 g fat mass; 38.3±4.7% adiposity) after maintenance on the HFD for approximately 7 months months. Prior to the onset of CL 316243 treatment on infusion day 7, both OT treatment groups were matched for OT-elicited reductions of weight gain.

**Figure 2A-E:**
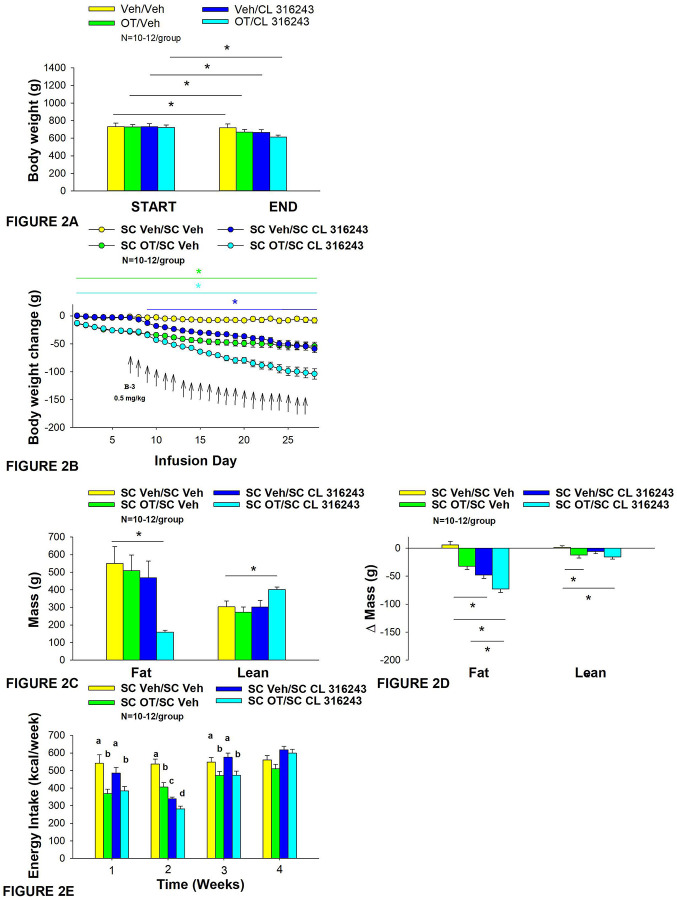
Effect of chronic 4V OT infusions (16 nmol/day) and systemic beta-3 receptor agonist (CL 31643) administration (0.5 mg/kg) on body weight, body adiposity and energy intake in male DIO rats. Ad libitum fed rats were either maintained on HFD (60% kcal from fat; N=8–10/group) for approximately 8 months prior to receiving continuous infusions of vehicle or OT (16 nmol/day) in combination with a single dose of CL 316243 (0.5 mg/kg). *A*, Change in body weight in HFD-fed DIO rats; *B*, Change in body weight gain in HFD-fed DIO rats; *C*, Change in fat mass and lean mass in HFD-fed DIO rats; *D*, Change in relative fat mass and lean mass in HFD-fed DIO rats; *E*, Weekly energy intake (kcal/week) in HFD-fed DIO rats. ↑ indicate 1x daily injections. Different letters denote significant differences between treatments *B*, Colored bars represent specific group comparisons vs vehicle. Data are expressed as mean ± SEM. *P<0.05, **P=0.05, †0.05<*P*<0.1 vs. vehicle or baseline (pre-treatment; Fig. 3A).

OT and CL 316243 alone reduced body weight by ≈ 7.8±1.3% (P<0.05) and 9.1±2.3% (*P*<0.05), respectively, but the combined treatment produced more pronounced weight loss (pre- vs post-intervention) (15.5±1.2%; *P*<0.05) (Figure 2A) than either treatment alone (*P*<0.05). OT alone tended to reduce weight gain on days 16–28 (0.05<*P*<0.1) while CL 316243 alone tended to reduce or reduced weight gain on day 24 (0.05<*P*<0.1), day 25 (*P*=0.05), and days 26–28 (*P*<0.05) (Figure 2B). OT and CL 316243 together tended to reduce weight gain on day 9 (0.05<*P*<0.1) reduced weight gain on days 10–28 (*P*<0.05). The combination treatment appeared to produce a more pronounced reduction of weight gain relative to OT alone on day 25 (0.05<P<0.1) and this reached significance on days 26–28 (*P*<0.05).

In addition, the combination treatment tended to produce a greater reduction of weight gain relative to CL 316243 alone on days 21–22 and 26–28 (0.05<*P*<0.1). While OT alone did not significantly reduce fat mass (*P*=NS), there was a tendency for CL 316243 alone (0.05<*P*<0.1), and the combination of OT and CL 316243 (*P*<0.05) to reduce fat mass without impacting lean body mass (Figure 2C; *P*=NS). However, the combination treatment did not result in a significant reduction of fat mass relative to OT alone or CL 316243 alone (*P*=NS). Consistent with these effects, OT alone did not produce a reduction in relative fat mass (*P*=0.108) while CL 316243 alone reduced relative fat mass (pre- vs post-intervention; *P*<0.05) without changing lean mass (Figure 2D; *P*=NS). The combination treatment produced a significant reduction of relative fat mass (pre- vs post-intervention; *P*<0.05) which exceeded that of OT alone (*P*<0.05) but not CL 316243 alone (*P*=NS). OT, CL 316243 and the combined treatment were all effective at reducing energy intake at week 2 (Figure 2E; *P*<0.05) with no difference between treatments (*P*=NS). All treatments were ineffective at reducing energy intake over weeks 3 and 4 (*P*=NS).

Two-way ANOVA revealed an overall significant effect of OT [(F(1,39) = 63.434, *P*<0.01)], CL 316243 [(F(1,39) = 74.939, *P*<0.01)] but no significant interactive effect between OT and CL 316243 [(F(1,39) = 0.058, *P*=NS)] on weight loss. Consistent with this finding, two-way ANOVA revealed consistent overall effects of OT and CL-3162343 on reduction of body weight gain between days 10–29 but no significant overall effect. In addition, two-way ANOVA revealed an overall significant effect of OT [(F(1,39) = 4.989, *P*=0.031)], CL 316243 [(F(1,39) = 7.607, *P*=0.009)] and a near significant interactive effect between OT and CL 316243 [(F(1,39) = 2.945, *P*=0.094)] on fat mass. There was no significant overall effect of OT [(F(1,39) = 1.351, *P*=NS)], on lean mass but there was an overall effect of CL 316243 [(F(1,39) = 4.829, *P*=0.034)], and an interactive effect of OT and CL 316243 [(F(1,39) = 4.905, *P*=0.033)] on lean mass.

Lastly, two-way ANOVA revealed an overall significant effect of OT [(F(1,39) = 21.464, *P*<0.01)], CL 316243 [(F(1,39) = 62.681, *P*<0.01)] and a near significant interactive effect between OT and CL 316243 [(F(1,39) = 3.190, *P*=0.082)] on energy intake (week 2).

Overall, these findings suggest an additive effect of OT and CL 316243 to produce sustained weight loss in DIO rats. The effects of the combination treatment on adiposity and energy intake appear to be driven largely by CL 316243 and OT, respectively.

CL 316243 elevated T_IBAT_ on injection day 1 at 0.5, 0.75 and 1-h post-injection (*P*<0.05; Table 2A) and tended to elevate T_IBAT_ at 0.25-h post-injection (0.05<*P*<0.1). Similarly, CL 316243, when given in combination with OT, also increased T_IBAT_ at 0.5, 0.75 and 1-h post-injection and tended to elevate T_IBAT_ at 0.25-h post-injection on injection day 1 (0.05<*P*<0.1). Both CL 316243 and CL 316243 + OT treatments elevated T_IBAT_ relative to vehicle treated animals when the T_IBAT_ data from injection day 1 were averaged over 1-h post-injection (TIBAT*P*<0.05). There was no significant difference in T_IBAT_ response to CL 316243 CL 316243 + OT treatments when the T_IBAT_ data were averaged over 60 min (*P*=NS).

CL 316243 also elevated T_IBAT_ on injection day 22 at 0.25, 0.5, 0.75 and 1-h post-injection (*P*<0.05; Table 2B). Similarly, CL 316243, when given in combination with OT, also increased T_IBAT_ at 0.5, 0.75 and 1-h post-injection and tended to elevate T_IBAT_ at 0.25 and 1-h post-injection on injection day 22 (0.05<*P*<0.1). Both CL 316243 and CL 316243 + OT treatments elevated T_IBAT_ relative to vehicle treated animals when the T_IBAT_ data from injection day 22 were averaged over 1-h post-injection (*P*<0.05). There was no significant difference in the T_IBAT_ response to CL 316243 and CL 316243 treatments when the T_IBAT_ data were averaged over 60 min (*P*=NS).

### Adipocyte size

**Figure 3A-H:**
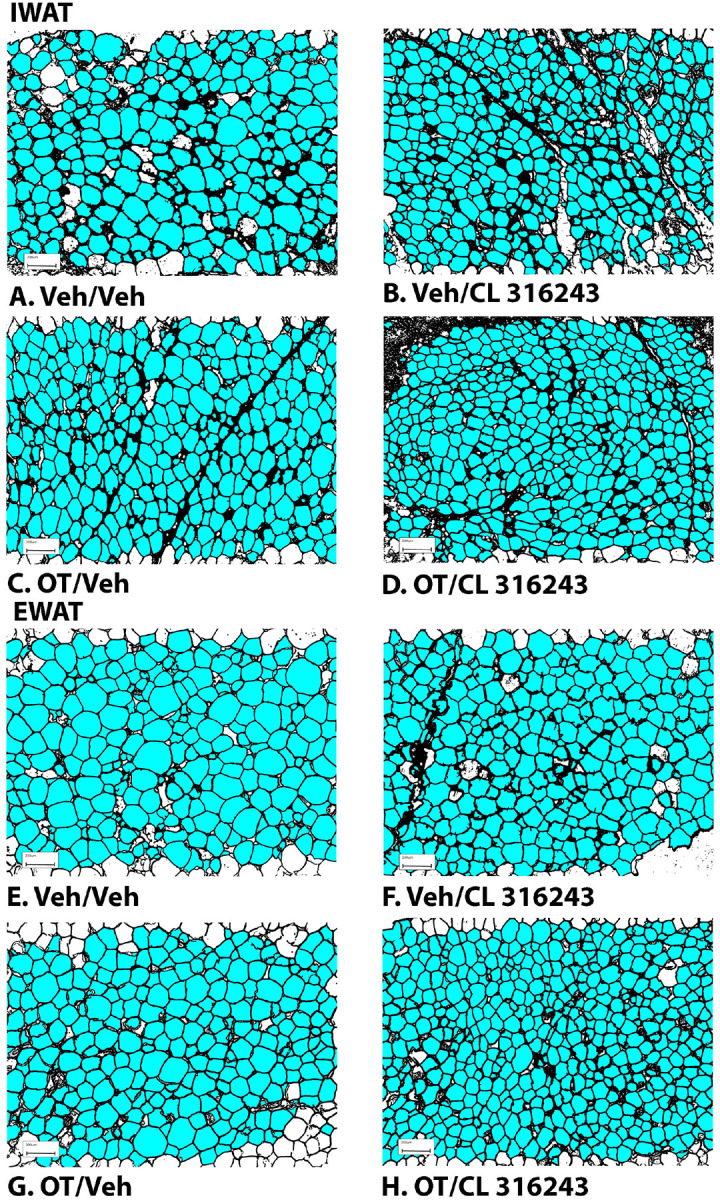
Effect of chronic 4V OT infusions (16 nmol/day) and systemic beta-3 receptor agonist (CL 31643) administration (0.5 mg/kg) on adipocyte size in IWAT and EWAT in male DIO rats. Adipocyte size was analyzed using ImageJ. Images were taken from fixed (4% PFA) paraffin embedded sections (5 μm) containing IWAT (A-D) or EWAT (E-H) in HFD-fed rats treated with 4V OT (16 nmol/day) or 4V vehicle in combination with IP CL 316243 (0.5 mg/kg) or IP vehicle. A/E, Veh/Veh. B/F, OT/Veh. C/G, Veh/CL 316243. D/H, OT-CL 316243; (A–H) all visualized at 100X magnification.

**Figure 4A-B:**
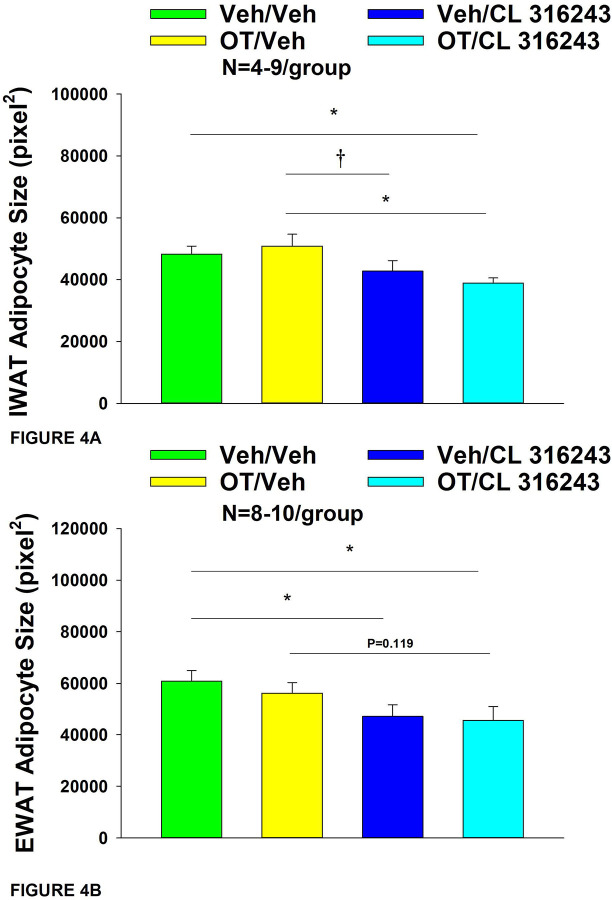
Effect of chronic 4V OT infusions (16 nmol/day) and systemic beta-3 receptor agonist (CL 31643) administration (0.5 mg/kg) on adipocyte size in IWAT and EWAT in male DIO rats. *A*, Adipocyte size (pixel^2^) was measured in IWAT from rats that received chronic 4V infusion of OT (16 nmol/day) or vehicle in combination with daily CL 316243 (0.5 mg/kg) or vehicle treatment (N=4–9/group). *B*, Adipocyte size was measured in EWAT from rats that received chronic 4V infusion of OT (16 nmol/day) or vehicle in combination with daily CL 316243 (0.5 mg/kg) or vehicle treatment (N=5–9/group). Data are expressed as mean ± SEM. *P<0.05 OT vs. vehicle.

OT and CL 316243 given in combination reduced IWAT adipocyte size whereas there was no significant effect of OT or CL 316343 on adipocyte size when given alone (*P*=NS) (Figure 3A–D; Figure 4A). In contrast, CL 316243 alone (*P*<0.05) or in combination with OT (*P*<0.05) reduced EWAT adipocyte size in DIO rats relative to vehicle treatment (**Figure 5E-H**; Figure 4B). There were no significant differences in the ability of the combined treatment to reduce EWAT adipocyte size relative to OT alone (*P*=0.119) or CL 316243 alone (*P*=0.156).

### Plasma hormone concentrations

To characterize the endocrine and metabolic effects of 4V OT (16 nmol/day) and systemic beta-3 receptor agonist (CL 316243) in DIO rats in a chronic study using as single dose of CL 316243 (**Study 2**; Table 3), we measured blood glucose levels and plasma concentrations of leptin, insulin, FGF-21, irisin, adiponectin, TC, triglycerides, and FFAs. CL 316243 alone or in combination with OT resulted in a reduction of plasma leptin relative to OT (*P*<0.05) or vehicle alone (*P*<0.05). The combination treatment was also associated with a reduction of blood glucose and insulin relative to vehicle (*P*<0.05) and OT treatment (*P*<0.05) but it was not statistically different from CL 316243 (*P*=NS). We also found that FGF-21 was reduced in response to CL 316243 and CL 316243 in combination with OT relative to vehicle and OT treatment (*P*<0.05). In addition, the combination treatment was associated with an elevation of adiponectin relative to vehicle (*P*<0.05) and OT treatment (*P*<0.05) but it was not statistically different from CL 316243 (*P*=NS).

### Gene expression data

We next determined the extent to which CL 316243 (0.5 mg/kg), OT (16 nmol/day), or the combination treatment increased thermogenic gene expression in IBAT relative to vehicle at 2-hour post-CL 316243/vehicle injections.

#### IBAT:

Consistent with published findings in mice and rats, chronic CL 316243 administration elevated relative levels of the thermogenic markers *Ucp1* [46; 47; 48], *Dio2* [34; 49], *Ppargc1a* [47] and *Gpr120* [34; 47] (Table 4A; *P*<0.05). CL 316243 in combination with OT also elevated *Ucp1*, *Dio2, Ppargc1a* and *Gpr120* (*P*<0.05). In addition, chronic CL 316243 alone and in combination with OT reduced *Adrb3* (β3-AR) mRNA expression in IBAT (Table 4A; *P*<0.05).

#### IWAT:

There were no significant differences in the thermogenic markers *Ucp1*, *Dio2*, *Ppargc1a* or *Adrb3* in response to chronic CL 316243 alone or in combination with OT (Table 4B; *P*=NS).

As a functional readout of BAT thermogenesis for the gene expression analyses, we measured T_IBAT_ in response to CL 316243 alone or CL 316243 + OT during the time period that preceded tissue collection. CL 316243 alone resulted in an increase in T_IBAT_ at 0.25, 0.5, 0.75 and 1-h post-injection (Table 5; *P*<0.05). Similarly, CL 316243 + OT resulted in an increase in T_IBAT_ at 0.25, 0.5 and 1-h post-injection (Table 5; *P*<0.05) and it also tended to increase T_IBAT_ at 0.75-h post-injection (Table 5; 0.05<*P*<0.1).

## Discussion

The goal of the current study was to determine the extent to which systemic OT could be used as an adjunct with the β3-AR agonist, CL 316243, to increase BAT thermogenesis and elicit weight loss in DIO rats. We hypothesized that systemic OT and beta-3 agonist (CL 316243) treatment would produce an additive effect to reduce body weight and adiposity in DIO rats by decreasing food intake and stimulating BAT thermogenesis. To test this hypothesis, we determined the effects of systemic (subcutaneous) infusions of OT (50 nmol/day) or vehicle (VEH) when combined with daily systemic (intraperitoneal) injections of CL 316243 (0.5 mg/kg) or VEH on body weight, adiposity, food intake and brown adipose tissue temperature (T_IBAT_). OT and CL 316243 monotherapy decreased body weight by 8.0±0.9% (*P*<0.05) and 8.6±0.6% (*P*<0.05), respectively, but OT in combination with CL 316243 produced more substantial weight loss (14.9±1.0%; *P*<0.05) compared to either treatment alone. These effects were associated with decreased adiposity, energy intake and elevated T_IBAT_ during the treatment period. In addition, systemic OT and CL 316243 combination therapy increased IBAT thermogenic gene expression suggesting that increased BAT thermogenesis may also contribute to these effects. The findings from the current study suggest that the effects of systemic OT and CL 316243 to reduce body weight and adiposity are additive and appear to be driven primarily by OT-elicited changes in food intake and CL 316243-elicited increases in BAT thermogenesis.

Recent findings also indicate that other agents, namely the glucagon-like peptide-1 receptor agonist, liraglutide, and the fat signal, oleoylethanolamide (OEA), act in an additive fashion with CL 316243 to reduce body weight or body weight gain. Oliveira recently reported that the combination of liraglutide and CL 316243 produce additive effects to reduce the change in body weight in a mouse model [50]. These effects appeared to be attributed to additive effects on energy intake and increased oxygen consumption in IBAT and IWAT. In addition, the combined treatment increased expression of UCP-1 in IWAT (indicative of browning). Similarly, Suarez and colleagues demonstrated that the peroxisome proliferator-activating receptor-α (PPARα) agonist and fat signal, oleoylethanolamide (OEA), act in an additive fashion with CL 316243 produced to reduce food intake and weight gain in rats [32]. These effects were associated with pronounced reductions in fat mass and increases in expression of thermogenic genes (*PPARα* and *Ucp1*) in EWAT [32]. Of interest is the finding that systemic OT and central OT can increase OEA expression in EWAT [19]. Furthermore, Deblon reported that the effectiveness of OT to decrease body weight was partially blocked in PPARα null mice [19], indicating that PPARα may partially mediate OT’s thermogenic effects in EWAT. OEA has also been found to stimulate 1) hypothalamic expression of OT mRNA [51], 2) PVN OT neurons [52], and 3) OT release within the PVN [52]. In addition, OEA also decreases food intake, in part, through OT receptor signaling [51]. Thus, it is possible that high fat diet-elicited stimulation of OEA [53] may reduce food intake, in part, through an OTR signaling and that the effects of OT to stimulate WAT thermogenesis might occur through PPARα. Additional studies that utilize adipose depot knockdown of PPARα will enable us to determine if PPARα in specific adipose depots may contribute to the ability of OT and CL 316243 to reduce body weight and adiposity.

Our findings and others raise the possibility that systemic OT could be reducing food intake and adiposity, in part, through a direct effect on peripheral OT receptors. Asker reported that OT-B12, a BBB-impermeable OT analogue, reduced food intake in rats, thus providing evidence that peripheral OTR signaling is important in the control of food intake [54]. Consistent with these findings, Iwasaki found that the ability of peripheral administration of OT to reduce food intake was attenuated in vagotomized mice [55; 56]. In addition, Brierley extended these findings and found that the effect of systemic administration of OT to suppress food intake required NTS preproglucagon neurons that receive direct synaptic input OTR-expressing vagal afferent neurons [57]. We also found that systemic administration of a non-BBB penetrant OTR antagonist, L-371257, stimulated food intake and body weight gain in [58]. Several studies have also found that subcutaneous infusion of OT reduced adipocyte size in 1) visceral fat in female Wistar rats that were peri- and postmenopausal [59] and 2) visceral fat in a dihydrotestosterone-elicited model of polycystic ovary syndrome in female Wistar rats [60], 3) subcutaneous fat in female ovariectomized Wistar rats [61], and 4) EWAT in male Zucker fatty rats [62]. More recent studies have confirmed that peripheral administration of long-acting OT analogues (including ASK2131 and ASK1476) also reduced both food intake and body weight [63; 64]. Together, these findings suggest that OT may also act in the periphery to decrease adipocyte size by a direct effect on OTRs found on adipose tissue [19; 20; 65]. Of translational importance is the finding that subcutaneous [19; 24; 25; 26; 27; 66] or intraperitoneal [27] administration of OT or long-acting OT analogues can recapitulate the effects of chronic CNS administration of OT on reductions of food intake and body weight.

The combination treatment and CL 316243 monotherapy reduced body weight and adiposity, in part, through increased BAT thermogenesis. Both CL 316243 alone and in combination with OT elevated T_IBAT_ throughout the course of the injection study and increased IBAT thermogenic genes (UCP-1, DIO2 and Ppargc1a) and UCP-1 content in IBAT. These findings coincided with CL- 316243-elicited increases in T_IBAT_ from the same animals during the time that preceded tissue collection. These findings are consistent with our previously published findings in rats [34] and other studies mice and rats that found chronic CL 316243 administration to increase the thermogenic markers DIO2 [49] and Gpr120 [46; 47; 48]. Similar to our findings, others also reported that systemic CL 316243 increased UCP-1 mRNA expression in mice [67]. We also found that the combination treatment and CL 316243 alone caused a downregulation of the β3-AR @ 2-h post-injection. This finding is consistent with we have previously reported [34] and others who have reported that both cold exposure and norepinephrine reduced β3-AR mRNA expression in mouse IBAT [68; 69] and.mouse brown adipocytes [70].

In summary, our results indicate that systemic administration of OT in combination with systemic CL 316243 treatment results in more profound reductions of body weight compared to either OT or CL 316243 alone. Moreover, the combined treatment of OT and CL 316243 stimulated BAT thermogenesis as determined by increased T_IBAT_ and thermogenic gene expression in IBAT. Together, our data support the hypothesis that systemic OT and β3-AR agonist (CL 316243) treatment produce an additive effect to reduce body weight and adiposity in DIO rats. The effects of the combined treatment on body weight and adiposity appeared to be additive and driven predominantly by OT-elicited reductions of food intake and CL-316243-elicited increases in BAT thermogenesis.

Collectively, these findings suggest that systemic OT treatment could be a viable adjunct to other anti-obesity treatment strategies. While intranasal OT has been found to reduce body weight by approximately 9.3% in a small study with limited subjects (≈9.3%) [71], it was not found, however, to have any effect on body weight in a larger scale clinical study (N=subjects) in which subjects were matched well for body weight, adiposity and gender [72]. Importantly, Plessow, Lawson and colleagues did find a significant effect of intranasal OT to reduce energy intake at 6-weeks post-treatment, which served as an important control. It is possible that a more extended length of treatment might have been required to take advantage of the reductions of energy intake that were not observed until the 6-week post-treatment time point. In addition, changes in dose, dosing frequency, or co-administration with Mg^2+^ [73; 74] might need to be taken into consideration in order to maximize the effects of intranasal OT on body weight in humans who are overweight or obese. Given that OT can be an effective delivery approach to reduce energy intake and elicit weight loss in several rodent models (see [15; 75; 76] for review) and obese nonhuman primates [18], it will also be important to determine if chronic systemic OT treatment can elicit weight loss when given in combination with CL 316243 at doses that are sub-threshold for producing adverse effects on heart rate or blood pressure [77]. Recent findings indicate that OT can be effective at reducing food intake and/or body weight in female rats [78] and DIO male mice [25], respectively. Thus, it will be important to examine if this combination treatment produces an additive effect to reduce body weight and adiposity in female DIO rodents and nonhuman primates.

## Figures and Tables

**Table 1.** Plasma measurements following systemic (SC) infusions of OT (16 and 50 nmol/day) or vehicle DIO rats. Data are expressed as mean ± SEM. *P<0.05 OT vs. vehicle (N=6–7/group).

SC	VEH	OT (16 nmol/day)	OT (50 nmol/day)
**Leptin (ng/mL)**	65.9 ± 7.8^a^	71.3 ± 8.2^a^	59.6 ± 7.7^a^
**Insulin (ng/mL)**	4.7 ± 1.7^a^	3.4 ± 0.9^a^	4.7 ± 1.3^a^
**Blood Glucose (mg/dL)**	137.3± 4.5^a^	160.4 ± 8.3^b^	157.7 ± 7.3^ab^
**Glucose (mg/dL)**	194.6 ± 5.7^a^	202.2 ± 8.0^a^	205 ± 12.5^a^
**Total Cholesterol (mg/dL)**	108.1 ± 10.4^a^	118.4 ± 13.4^a^	105.5 ± 11.5^a^

Different letters denote significant differences between treatments

Shared letters are not significantly different from one another

N=6–7/group

**Table 2A-C.** T_IBAT_ measurements following acute systemic administration of the beta-3 receptor agonist (CL 316243) or vehicle in male DIO rats. *A*, injection day 1 (0.5 mg/kg) and *B*, injection day 22 (0.5 mg/kg). Data are expressed as mean ± SEM. *P<0.05 vs VEH; †0.05<*P*<0.1 vs VEH.

Table 2A. Changes in T_IBAT_ Following Systemic OT +/− CL 316243						
Treatment in Male DIO Rats (Injection Day 1)						
SC	IP	0 min	15 min	30 min	45 min	60 min
		Temp (C°)	Temp (C°)	Temp (C°)	Temp (C°)	Temp (C°)
**VEH**	**VEH**	37.1±.0.4	37.3±.0.8	37.7±.0.3	37.5±.0.3	37.4±.0.3
**VEH**	**CL 316243**	36.8±.0.2	38.3±.0.2†	38.6±.0.2*	38.5±.0.2*	38.5±.0.2*
**OT**	**VEH**	36.6±.0.3	37.7±.0.2	37.3±.0.2	37.3±.0.2	37.0±.0.2
**OT**	**CL 316243**	36.6±.0.2	38.2±.0.2†	38.4±.02*	38.3±.0.2*	38.1±.0.1*
*P<0.05 vs VEH						
**N=8–12/group**						
Table 2B. Changes in T_IBAT_ Following Systemic OT +/− CL 316243						
Treatment in Male DIO Rats (Injection Dav 22)						
SC	IP	0 min	15 min	30 min	45 min	60 min
		Temp (C°)	Temp (C°)	Temp (C°)	Temp (C°)	Temp (C°)
**VEH**	**VEH**	36.5±.0.3	37.7±.0.2	37.1±.0.3	37.0±.0.2	37.1±.0.3
**VEH**	**CL 316243**	36.8±.0.2	38.5±.0.2*	38.3±.0.2*	38.2±.0.2*	38.1±.0.2*
**OT**	**VEH**	36.2±.0.2	37.4±.0.1	37.0±.0.2	36.9±.0.2	37.0±.0.2
**OT**	**CL 316243**	36.4±.0.1	38.1±.0.2†	37.9±.0.1*	37.7±.0.2*	37.7±.0.2†
*P<0.05 vs VEH						
**N=10–12/group**						

**Table 3.** Plasma measurements following 4V infusions of OT (16 nmol/day), CL 316243 (0.5 mg/kg) or OT (16 nmol/day) + CL 316243 (0.5 mg/kg) in DIO rats. Data are expressed as mean ± SEM. Different letters denote significant differences between treatments (*P*<0.05).

SC	VEH/VEH	OTA/VEH	VEH/CL 316243	OT/CL 316243
**Leptin (ng/mL)**	47.2 ± 6.0^a^	45.9 ± 4.9^a^	25.8 ± 3.4^b^	22.0 ± 2.4^b^
**Insulin (ng/mL)**	10.9 ± 2.3^ac^	11.7 ± 1.5^a^	6.6 ± 1.2^bc^	4.3 ± 0.8^b^
**FGF-21 (pg/mL)**	187.3 ± 22.5^a^	163.9 ± 9.1^a^	87.7 ± 8.0^b^	101.5 ± 11.6^c^
**Irisin (μg/mL)**	3.9 ± 0.3^a^	4.5 ± 0.6^a^	4.6 ± 0.4^a^	4.4 ± 0.4^a^
**Adiponectin (μg/mL)**	6.4 ± 0.6^ac^	6.3 ± 0.6^ac^	7.3 ± 0.6^bc^	8.7 ± 0.5^b^
**Blood Glucose (mg/dL)**	179.3 ± 24.5^a^	174.9 ± 9.3^a^	150 ± 3.7^ab^	138 ± 4.5^b^
**Glucose (mg/dL)**	206.2 ± 10.7^a^	213.1 ± 12.2^a^	173.2 ± 7.7^b^	180.9 ± 6.3^b^
**Triglycerides (mg/dL)**	81.5 ± 13.7^ab^	97.5 ± 33.1^a^	39.8 ± 3.9^b^	47.8 ± 3.3^ab^
**FFA (mEq/L)**	0.5 ± 0.1^a^	0.5 ± 0.1^a^	0.2 ± 0.02^b^	0.3 ± 0.03^ab^
**Total Cholesterol (mg/dL)**	100.0 ± 6.0^a^	108.1 ± 9.7^a^	94.5 ± 5.1^a^	107.0 ± 10.2^a^

Different letters denote significant differences between treatments

Shared letters are not significantly different from one another

N=10–12/group

**Table 4.** Changes in IBAT mRNA expression following chronic 4V OT (16 nmol/day) and systemic CL 316243 (0.5 mg/kg) treatment in male DIO rats. Data are expressed as mean ± SEM. Different letters denote significant differences between treatments (*P*<0.05).

Table 4A. Changes in IEAT mRNA Expression Following SC OT and CL 316243 Treatment in Male				
DIO Rats				
SC Treatment	VEH/VEH	OT/VEH	VEH/CL 316243	OT/CL 316243
**IBAT**				
*Adrb1*	1.0 ± 0.3^a^	1.0 ± 0.1^a^	0.8 ± 0.1^a^	0.9 ± 0.4^a^
*Adrb3*	1.0 ± 0.1^a^	0.9 ± 0.2^a^	0.5 ± 0.1^b^	0.5 ± 0.04^b^
*Ucp1*	1.0 ± 0.1^a^	1.0 ± 0.1^a^	1.6 ± 0.1^b^	1.6 ± 0.1^b^
*Cidea*	1.0 ± 0.1^a^	1.0 ± 0.1^a^	1.0± 0.1^a^	0.9 ± 0.1^a^
*Dio2*	1.0 ± 0.1^a^	1.5 ± 0.2^a^	3.3 ± 0.4^b^	3.5 ± 0.3^b^
*Gpr120*	1.0 ± 0.2^a^	0.9 ± 0.2^a^	19.8 ± 2.4^b^	15.9 ± 2.1^b^
*Prdm16*	1.0 ± 0.1^a^	0.9 ± 0.1^a^	0.8 ± 0.1^a^	0.9 ± 0.1^a^
*Ppargc1a*	1.0 ± 0.05^a^	1.0 ± 0.2^a^	4.8 ± 0.4^b^	5.9 ± 0.5^c^
**Different letters denote significant differences between treatments**				
**Shared letters are not significantly different from one another**				
**N=10–12/group**				
Table 4B. Changes in IWAT mRNA Expression Following SC OT and CL 316243 Treatment in Male				
DIO Rats				
SC Treatment	VEH/VEH	OTA/VEH	VEH/CL 316243	OT/CL 316243
**IWAT**				
*Adrb3*	1.0 ± 0.4^a^	1.1 ± 0.6^a^	1.0 ± 0.3^a^	0.5 ± 0.2^a^
*Ucp1*	1.0 ± 0.4^a^	0.3 ± 0.1^a^	0.5 ± 0.2^a^	1.7 ± 0.8^a^
*Dio2*	1.0 ± 0.3^a^	1.0 ± 0.2^a^	0.6 ± 0.2^a^	0.9 ± 0.3^a^
*Ppargc1a*	1.0 ± 0.7^a^	0.5 ± 0.4^a^	1.5 ± 1.0^a^	0.04 ± 0.02^a^
**Different letters denote significant differences between treatments**				
**Shared letters are not significantly different from one another**				
**N =9–12/group**				

**Table 5.** T_IBAT_ measurements following acute systemic administration of the beta-3 receptor agonist (CL 316243) or vehicle in male DIO rats. *A*, injection day 23 prior to euthanasia (0.5 mg/kg), euthanasia day. Data are expressed as mean ± SEM. *P<0.05 vs VEH; †0.05<*P*<0.1 vs VEH.

SC	IP	0 min	15 min	30 min	45 min	60 min
		**Temp (C°)**	**Temp (C°)**	**Temp (C°)**	**Temp (C°)**	**Temp (C°)**
**VEH**	**VEH**	36.7±.0.3	37.5±.0.2	37.4±.0.2	37.5±.0.2	37.2±.0.7
**VEH**	**CL 316243**	36.7±.0.1	38.3±.0.3*	38.5±.0.2*	38.5±.0.2*	38.3±.0.2*
**OT**	**VEH**	36.3±.0.1	37.1±.0.3	37.5±.0.2	37.5±.0.2	37.1±.0.3
**OT**	**CL 316243**	36.7±.0.2	37.8±.0.2*	38.1±.0.2*	38.0±.0.2†	37.8±.0.2*

* P<0.05 vs VEH

† 0.05<P<0.1

N=9–12/group
