# Supplementary material for: Effects of systemic oxytocin and beta-3 receptor agonist (CL 316243) treatment on body weight and adiposity in male diet-induced obese rats
Source: bioRxiv. 2025 Jan 27:2024.09.27.615550. Originally published 2024 Sep 30. Preprint. [Version 3] doi: 10.1101/2024.09.27.615550 (PMC11537314; doi:10.1101/2024.09.27.615550)
Supplement: Supplement 1 [file NIHPP2024.09.27.615550v3-supplement-1.pdf]

bioRxiv preprint doi: https://doi.org/10.1101/2024.09.27.615550; this version posted January 27, 2025. The copyright holder for this preprint (which was not certified by peer review) is the author/funder. This article is a US Government work. It is not subject to copyright under 17 USC 105 and is also made available for use under a CC0 license.

Supplemental Figure 1A-B: T_{IBAT} measurements following acute systemic administration of the beta-3 receptor agonist (CL 316243) or vehicle in male DIO rats. *A*, injection day 1 (0.5 mg/kg) and *B*, injection day 22 (0.5 mg/kg). Data are expressed as mean ± SEM. *P<0.05 vs VEH; †0.05<*P*<0.1 vs VEH.

Supplemental Figure 2A-H: Representative image to illustrate the effect of chronic systemic OT infusions (50 nmol/day) and systemic beta-3 receptor agonist (CL 316243) administration (0.5 mg/kg) on adipocyte size in EWAT and IWAT in male DIO rats. Adipocyte size was analyzed using ImageJ. Images were taken from fixed (4% PFA) paraffin embedded sections (5 µm) containing EWAT (A-D) or IWAT (E-H) in HFD-fed rats treated with systemic OT (50 nmol/day) or vehicle in combination with IP CL 316243 (0.5 mg/kg) or IP vehicle. A/E, Veh/Veh. B/F, OT/Veh. C/G, Veh/CL 316243. D/H, OT-CL 316243; (A–H) all visualized at 100X magnification.

Supplemental Figure 3. T_{IBAT} measurements following acute systemic administration of the beta-3 receptor agonist (CL 316243) or vehicle in male DIO rats. *A*, injection day 23 prior to euthanasia (0.5 mg/kg), euthanasia day. Data are expressed as mean ± SEM. *P<0.05 vs VEH; †0.05<*P*<0.1 vs VEH.

bioRxiv preprint doi: https://doi.org/10.1101/2024.09.27.615550; this version posted January 27, 2025. The copyright holder for this preprint (which was not certified by peer review) is the author/funder. This article is a US Government work. It is not subject to copyright under 17 USC 105 and is also made available for use under a CC0 license.

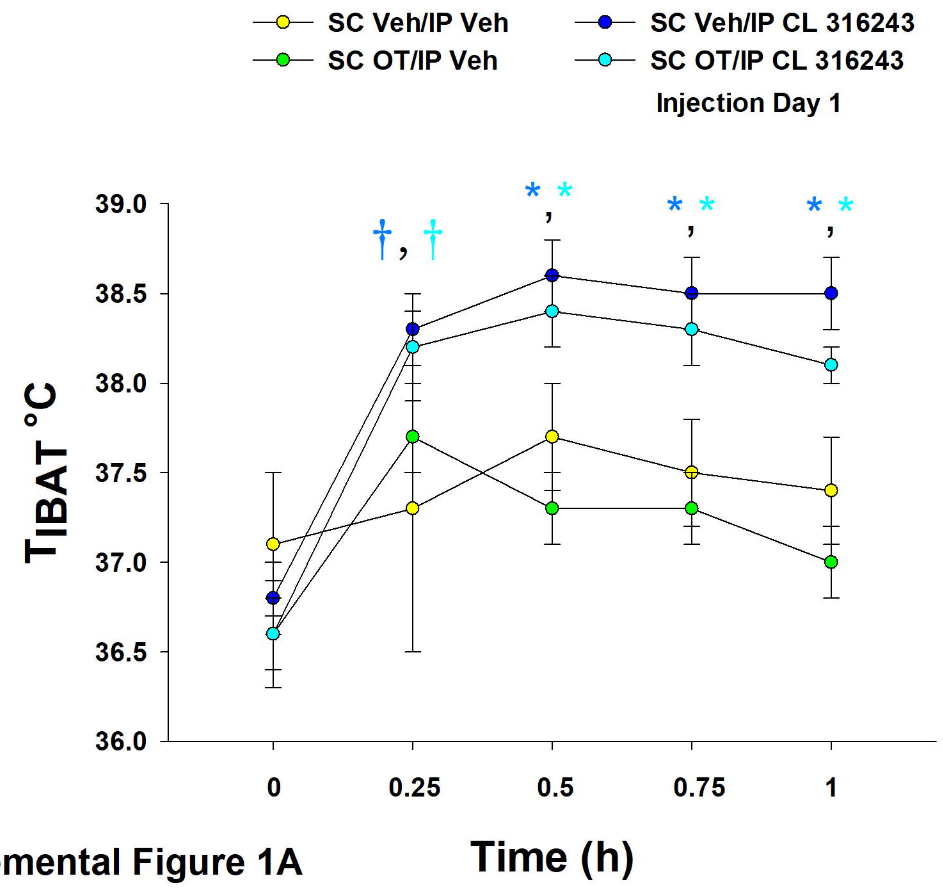

Supplemental Figure 1A

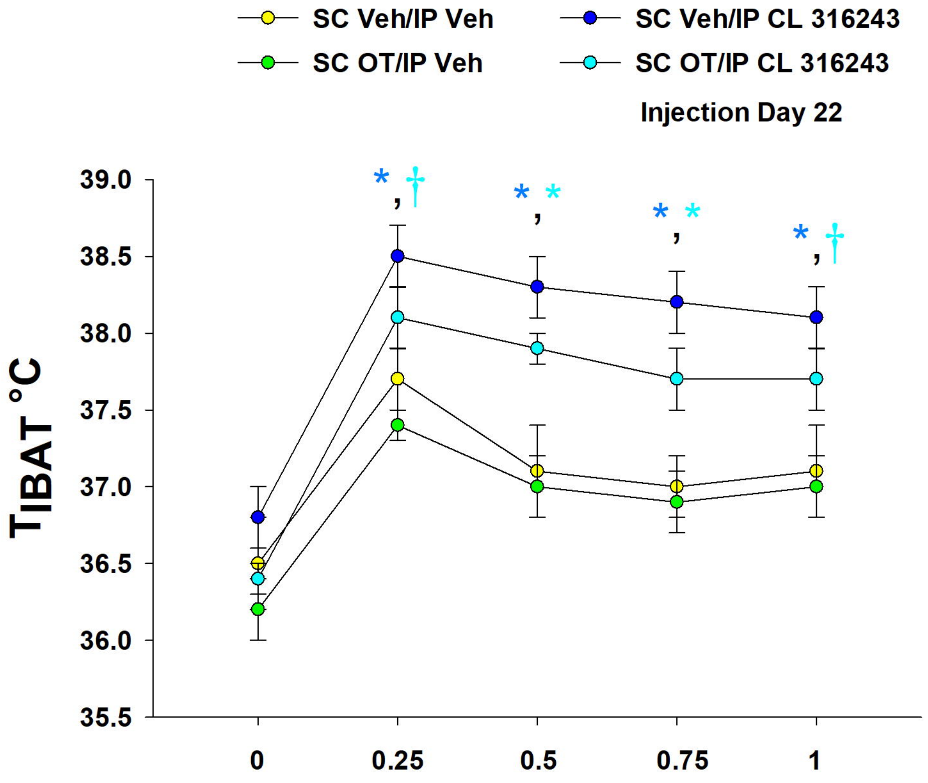

Supplemental Figure 1B

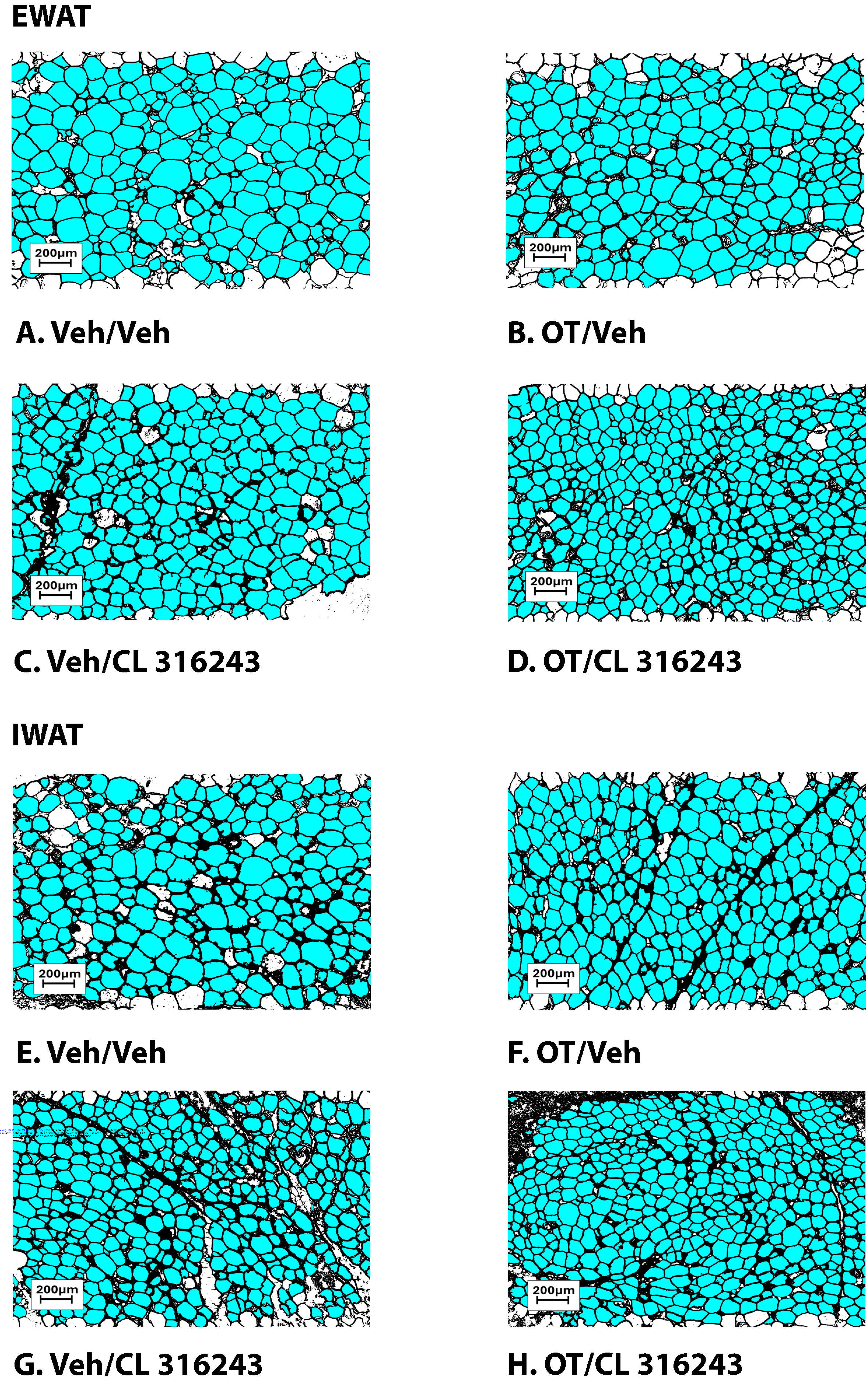

Supplemental Figure 2

bioRxiv preprint doi: https://doi.org/10.1101/2024.09.27.615550; this version posted January 27, 2025. The copyright holder for this preprint (which was not certified by peer review) is the author/funder. This article is a US Government work. It is not subject to copyright under 17 USC 105 and is also made available for use under a CC0 license.

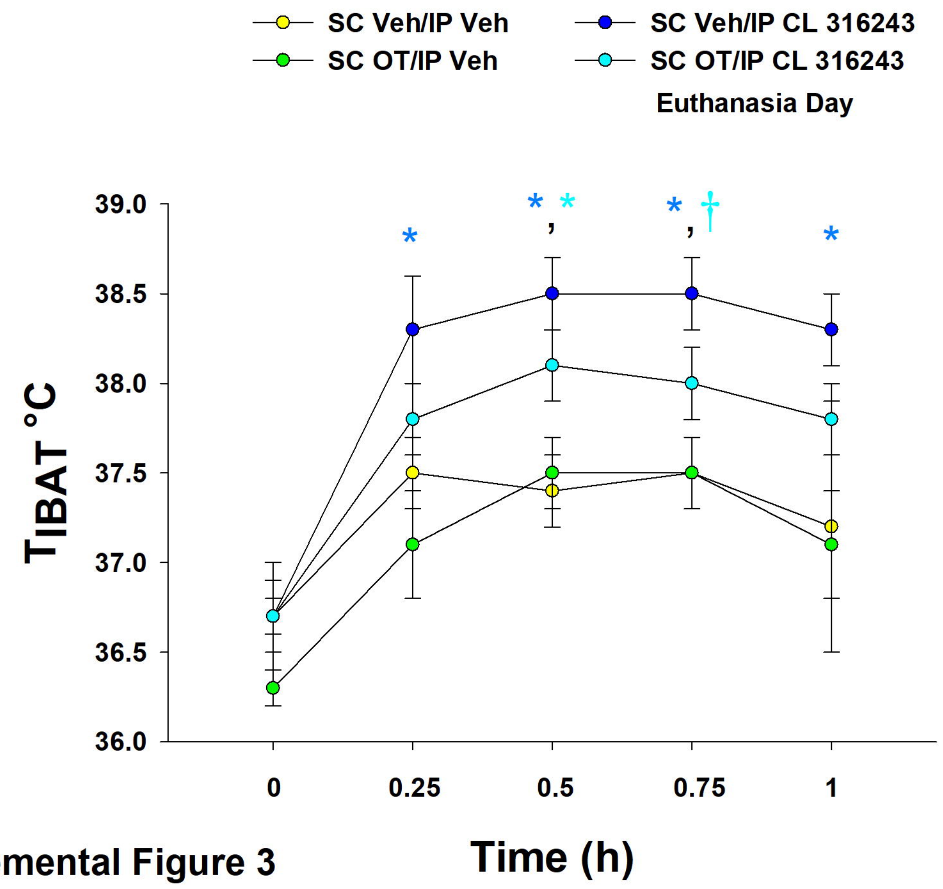

Supplemental Figure 3
